# Tisagenlecleucel yields superior patient-reported health-related quality of life compared to autologous stem cell transplantation in patients with relapsed/refractory large B-cell lymphomas

**DOI:** 10.1007/s00277-026-06840-5

**Published:** 2026-02-05

**Authors:** Ellen Obstfelder, Johannes Herrmann, Vladan Vučinić, Andreas Hochhaus, Ulf Schnetzke, Farina Eigendorff

**Affiliations:** 1 Klinik für Innere Medizin II, Abteilung für Hämatologie und Internistische Onkologie, Universitätsklinikum Jena, Am Klinikum 1, 07747 Jena, Germany; 2 Comprehensive Cancer Center Central Germany, Campus Jena, Jena, Germany; 3 Klinik und Poliklinik für Hämatologie, Zelltherapie, Hämostaseologie und Infektiologie, Universitätsklinikum Leipzig, Leipzig, Germany; 4 Comprehensive Cancer Center Central Germany, Campus Leipzig, Leipzig, Germany; 5 Fraunhofer Institute for Celltherapy and Immunology, Leipzig, Germany

**Keywords:** CAR T, Large B-cell lymphoma, Tisagenlecleucel, Patient-reported outcome, Health-related quality of life

## Abstract

Chimeric Antigen Receptor (CAR) T-cell therapy is an established treatment for relapsed or refractory large B-cell lymphoma (rr LBCL). While clinical efficacy is well documented, data on health-related quality of life (HRQoL) remain limited. This study assessed HRQoL in rr LBCL patients with long-term remission after CAR T-cell therapy versus high-dose chemotherapy with autologous stem cell transplantation (HD-ASCT). Twenty-eight consecutive rr LBCL patients in sustained remission were analyzed, with 15 receiving CAR T-cell therapy (tisagenlecleucel) and 13 undergoing HD-ASCT between 2019 and 2023. HRQoL was assessed using EQ-5D-5 L and PROMIS-29 questionnaires at 12 months and at a median of 36.8 months (range 15.7–57.2 months) post cellular therapy. Groups were compared for differences in HRQoL indices and domain scores. Median age was 63.5 (range 23–73) years. Twelve months post-treatment, CAR T-cell recipients reported significantly higher HRQoL scores than HD-ASCT patients (EQ-5D-5 L index value: 0.89 ± 0.11 vs. 0.72 ± 0.21; *p* = 0.015). Consistently, PROMIS-29 revealed clinically meaningful advantages in the CAR T-cell cohort across all seven categories. At later follow-up, EQ-5D-5 L index values converged (index-value 0.82 ± 0.22 vs. 0.7 ± 0.25; *p* = 0.2), whereas PROMIS-29 still indicated sustained benefit in five domains among CAR T-cell recipients. CAR T-cell therapy was associated with superior HRQoL in the first year compared to HD-ASCT. Although some differences diminished over time, CAR T-cell patients continued to experience better outcomes in several domains. These findings highlight clinical relevance of patient-reported outcome in rr LBCL, particularly in longitudinal surveys, and underscore the value of integrating patient-centered metrics into therapeutic decisions-making.

**Supplementary Information:**

The online version contains supplementary material available at 10.1007/s00277-026-06840-5.

## Introduction

Relapsed/refractory large B-cell lymphoma (rr LBCL) represents a significant therapeutic challenge, often characterizing a patient population with poor prognosis. Chimeric Antigen Receptor T-cell (CAR T) therapy has significantly altered the treatment landscape, introducing a potentially curative option for these patients. Tisagenlecleucel (tisa-cel, Kymriah^®^) was the first CD19 directed CAR T-cell therapy approved for use in children and young adults with relapsed or refractory acute lymphoblastic leukemia (ALL) by the US Food and Drug Administration (FDA) in 2017 [1]. In 2018, tisa-cel was approved both by the FDA and European Medicines Agency (EMA) for treatment of adult patients with LBCL refractory to ≥ 2 prior lines of therapy [2, 3]. However, in the second treatment line, higher response rates and progression-free survival (PFS) have been demonstrated for CAR T-cell products axicabtagene cileleucel (axi-cel) [4] and lisocabtagene macaleucel (liso-cel) [5] but not for tisa-cel [6] in phase 3 trials comparing this approach to high-dose therapy followed by autologous stem cell transplantation (HD-ASCT). Nonetheless, HD-ASCT remains standard treatment for suitable patients with late rr LBCL (> 12 months).

The impact of patient-reported health-related quality of life (HRQoL) as a measure of clinical effectiveness in a uniquely patient-centric manner has been increasingly recognized [7], but HRQoL data in patients with rr LBCL receiving cellular therapies remain limited. Recommended time points for patient reported outcomes (PRO) after cellular therapies are divided into three phases: acute (up to 30 days after therapy), sub-acute (up to 12 months after therapy) and long term (up to 5 years after therapy) [8]. To date, most studies have focused on the acute and subacute periods, with far-less long-term survivors [9]. This highlights a substantial knowledge gap in the late post-treatment phase.

Emerging evidence suggests that CAR T-cell therapy can offer meaningful quality-of-life benefits in responding patients. A recent systematic review and meta-analysis which pooled 15 studies, concluded that CAR T-cell treatment led to clinically significant improvements in at least six major HRQoL domains [10]. PRO analyses from pivotal trials further illustrate this: in the phase 2 JULIET study, patients achieving remission showed sustained and clinically meaningful improvements in HRQoL compared to baseline over a follow-up of 19 months [11]. Similar results were also shown for patients treated with axi-cel 90 days after infusion, representing patients’ recovery from acute toxicity phase including cytokine release syndrome (CRS) and immune effector cell-associated neurotoxicity (ICANS) [12]. The phase 3 ZUMA-7 trial comparing second-line axi-cel to standard chemotherapy/HD-ASCT showed statistically and clinically superior HRQoL for CAR T-cell recipients at day 100 and day 150 post-therapy, persisting through 24 months of follow-up [13]. Superiority of longitudinally assessed HRQoL was also shown for CAR T-cell therapy compared to HD-ASCT, or allogeneic SCT within the first 6 months albeit in a very heterogeneous disease population [14].

In summary, prior research indicates that CAR T-cell therapy can improve HRQoL in responding patients, especially after the acute phase, whereas HD-ASCT is associated with well-known chemotherapy-related quality-of-life detriments [15]. Nevertheless, comprehensive long-term PRO comparisons between CAR T-cell therapy and HD-ASCT in a uniformly responding patient population are lacking. Here, we present a long-term follow-up study examining HRQoL in rr LBCL survivors who underwent either CAR T-cell therapy (tisa-cel) or HD-ASCT. By evaluating two time points (1 year and ~ 3 years post treatment) in patients who achieved a remission, we isolate the impact of treatment modality in quality of life, minimizing confounding by active disease.

## Patients and methods

### Patient cohort and informed consent

This cohort included 28 consecutive adult patients treated for rr LBCL with either tisa-cel or HD-ASCT from September 2019 to April 2023 at Jena University Hospital, who achieved long-term remission. Fifteen patients received tisa-cel, and 13 patients underwent HD-ASCT. Patients were treated in the setting of the European Medicine Agency (EMA) approval label: CAR T-cell therapy was administered for rr LBCL after at least two prior lines of treatment. Patient information was documented in the European Society for Blood and Marrow Transplantation (EBMT)/DRST database. This study was approved by the local Ethics Committee (2023-3166-Reg) and performed in accordance with the Declaration of Helsinki.

### Data collection and patient-reported HRQoL assessments

Clinical data were abstracted from each patient’s chart in the institutional database. HRQol was assessed using EuroQoL 5-Dimension 5-Level (EQ-5D-5 L) and the Patient-Reported Outcome Measurement Information System 29-item profile (PROMIS-29 v.2.1) due to their predominant use as PROs related to CAR T-cell therapy [9]. This dual selection enabled comprehensive evaluation of HRQoL by combining the country-specific reference of the EQ-5D-5 L with the detailed domain-specific assessment capabilities of the PROMIS-29. Although originally validated in the general US population, the PROMIS-29 has been culturally adapted and psychometrically validated for Germany, enabling a reliable assessment within our cohort [16]. Assessments were completed via paper questionnaires and conducted at two time points. The first time point was conducted 12 months post therapy and the second time point at a median of 36.8 months (range 15.7–57.2) post-therapy.

### EQ-5D-5L

The EQ-5D-5L is a standardized instrument developed by the EuroQoL Group, designed to provide a simple, generic measure of health for clinical and economic appraisal. It comprises five dimensions: Mobility, Self-Care, Usual Activities, Pain/Discomfort, and Anxiety/Depression. The valuation of the EQ-5D-5L responses was conducted using the crosswalk index value calculator provided by the EuroQoL Group. This method translates the respondent’s status across the five dimensions into a single summary index by applying country-specific value sets, which reflect the preferences of the general population of that country regarding different health states [17]. In this study, we applied the valuation set for Germany to convert the EQ-5D-5L health states into utility values. These utility values range from − 0.661 to 1, where 1 represents perfect health, 0 represents death, and negative values represent health states perceived as worse than death [18]. Moreover, the EQ Visual Analogue Scale (EQ-VAS) was employed. The VAS is a standard instrument for recording an individual’s rating of their current HRQoL on a vertical visual analogue scale, where the endpoints are labeled ‘The best health you can imagine’ and ‘The worst health you can imagine’. This provides a direct quantification of the patient’s health as perceived by the individual themselves, ranging from 0 to 100 [17].

### PROMIS-29

The PROMIS-29 v2.1 is a well-validated, self-administered instrument comprising 29 items that provide a comprehensive assessment across seven domains: Physical Function, Anxiety, Depression, Fatigue, Sleep Disturbance, Social Participation, and Pain Interference. Each domain includes four questions, except for pain interference, which has an additional item assessing pain intensity on a 0 to 10 scale, from no pain to worst imaginable pain (numeric rating scale, NRS). Patients responded to each item using a.

5-point Likert scale, where higher scores indicate greater impairment or more severe symptoms, except in the domain of physical function, where higher scores indicate better function. The raw scores for each domain were converted to T-scores using the *HealthMeasures Scoring Service* [19] and algorithms [20].

These T-scores have a mean of 50 and a standard deviation of 10 in the general population of the United States. A greater T-score on the PROMIS scale reflects an increased presence of the attribute being assessed. In the context of symptom dimensions, which encompass Anxiety, Depression, Fatigue, Sleep Disturbance, and Pain, a superior score signifies more severe symptoms (normal 20–55, mild 55–60, moderate 60–70, severe 70–80). Conversely, within the functional areas, such as physical function and social participation, a superior score denotes enhanced functioning (normal 45–80, mild 40–45, moderate 30–40, severe 20–30) [21]. Differences of ≥ 3 T-Score points were interpreted as clinically significant based on standard conventions in PROMIS scoring.

### Safety analyses

Hematological toxicity for all patients was defined as followed: Severe neutropenia was characterized by an absolute neutrophil count (ANC) < 500/µl, severe thrombocytopenia by platelet counts < 20/nL and/or requiring transfusions, and severe anemia by hemoglobin levels < 8 g/dL and/or requiring transfusions. Long-term hematotoxicity following CAR T-cell therapy included prolonged neutropenia (defined by ANC < 1000/µl and/or G-CSF dependence lasting ≥ 21 days after CAR T-cell infusion and continuing for ≥ 21 days), prolonged thrombocytopenia (defined by platelet counts < 20/nL and/or requiring transfusions measured ≥ 21 days after CAR T-cell infusion and continuing for ≥ 21 days), and prolonged anemia (defined by hemoglobin < 8 g/dL and/or requiring transfusions measured ≥ 21 days after CAR T-cell infusion and continuing for ≥ 21 days).

### Statistics

Group comparisons used Mann-Whitney-U-test for patient characteristics and hematotoxicity. Fisher exact test was applied for patient characteristics with small sample sizes below five to assess differences between groups. The EQ-5D-5 L index score was considered analyzable if answers were provided for all five dimensions. Data were analyzed using descriptive statistics to summarize the EQ-5D-5 L health profile, describing the number and percentage of patients reporting each level (level 1: no problems; level 2: slight problems; level 3: moderate problems; level 4: severe problems; level 5: extreme problems/unable to do) on each dimension. Chi-square test was used to analyze the distribution of EQ-5D-5 L dimension responses at baseline and at follow-up, and between both patient groups. For cross-sectional comparisons between the two populations at each time point, independent t- test was used for the index value and EQ-VAS score, and paired t-test in the longitudinal analysis. For classification within the general population PROMIS-29 T-score cut points were used as described above. For the analysis of the PROMIS-29 scores, we conducted independent t-test to evaluate notable differences across the groups and paired t-test to assess differences in the longitudinal analysis. All tests were two sided with level of significance of 0.05. Analyses were performed by SPSS 30.0 (SPSS Inc., Chicago, IL, USA).

## Results

### Patients/Baseline characteristics

A total of 28 patients were included, comprising 15 patients who received CAR T-cell therapy and 13 who underwent HD-ASCT. The median age at treatment initiation was significantly higher in the CAR T-cell cohort than in the HD-ASCT cohort (65 years, interquartile range (IQR) 5.5 vs. 57 years, IQR 23; *p* = 0.019). The CAR T-cell group included 4 females (27%) and 11 males (73%), while the HD-ASCT group consisted of 9 females (69%) and 4 males (31%). Eastern Cooperative Oncology Group performance status (ECOG PS) at treatment initiation was 1 (IQR 1) in CAR T-cell recipients and 0 (IQR 1) in HD-ASCT recipients. Large B-cell lymphoma not otherwise specified (LBCL, NOS) was the most frequent lymphoma entity, occurring in 10 CAR T-cell patients (67%) and 8 HD-ASCT patients (62%), respectively.

CAR T-cell recipients had significantly more prior lines of therapy (median 4 vs. 2; *p* < 0.001) and almost half (*n* = 7; 47%) had undergone a prior HD-ASCT before eventually receiving CAR T-cell therapy, indicating a more heavily pretreated population (Table 1).

**Table 1:** Patient characteristics

	Category	CAR T	HD-ASCT	*p*-value
Age at therapy (years), median (IQR)	-	65 (5.5)	57 (23)	0.019
Sex, n (%)	Female	4 (27)	9 (69)	0.056
	Male	11 (73)	4 (31)	
ECOG PS at therapy, median (IQR)	-	1 (1)	0 (1)	0.170
Lymphoma entity, n (%)	LBCL, NOS	10 (67)	8 (62)	n.a.
	HGBL with MYC and BCL2 and/or BCL6 rearrangement	2 (13)	-	
	tLBCL	2 (13)	2 (15)	
	SCNSL	1 (7)	2 (15)	
	PMBCL	-	1 (8)	
Cell of origin (molecular subgroup), n (%)	GCB	5 (33.3)	3 (23.1)	n.a.
	ABC	3 (20.0)	2 (15.4)	
	n.d.	2 (13.3)	3 (23.1)	
Prior lines of therapies, median (IQR)	-	4 (1)	2 (0)	< 0.001
Prior HD-ASCT, n (%)	Yes	7 (47)	-	n.a.
	No	8 (53)		
HD-protocol, n (%)	R-TEAM	1 (11)	7 (54)	n.a.
	R-BEAM	4 (45)	-	
	(R)-Treosulfan/Fludarabine	2 (22)	5 (38)	
	other	2 (22)	1 (8)	
IPI at CAR T-cell/HD-ASCT therapy, n (%)	0–2 (low/low-intermediate)	6 (40)	5 (38)	0.670
	3–5 (high-intermediate/high)	6 (40)	3 (24)	
	n.d.	3 (20)	5 (38)	
LDH at CAR T-cell/HD-ASCT therapy, n (%)	≤ ULN	9 (60)	10 (77)	0.435
	≥ ULN	6 (40)	3 (33)	
CRS, n (%)	1	5 (33)	-	n.a.
	2	3 (20)		
	3	6 (40)		
	4	-		
	No CRS	1 (7)		
ICANS, n (%)	1	2 (13)	-	n.a.
	2	-		
	3	1 (7)		
	4	-		
	No ICANS	12 (80)		
Infectious complications during hospital stay, n (%)	Yes	3 (20)	12 (92)	< 0.001
	No	12 (80)	1 (8)	
Other complications, n (%)	Mucositis oral (CTCAE grade ≥ 3)	-	7 (50)	n.a.
	Diarrhea		4 (29)	
	Skin toxicity		2 (14)	
	other		1 (7)	
Hospitalization time (days), median (IQR)	-	22 (8.5)	29 (10)	0.065

Abbreviations: *ECOG PS* eastern cooperative oncology group performance status, *LBCL NOS* large B-cell lymphoma not otherwise specified, *HGBL* high-grade B-cell lymphoma, *tLBCL* transformed large B-cell lymphoma, *SCNSL* secondary central nervous system lymphoma, *PMBCL* primary mediastinal B-cell lymphoma, *GCB* germinal center B-cell like, *ABC* activated B-cell like, *IPI* international prognostic index, *IQR* interquartile range, *LDH* lactate dehydrogenase, *ULN* upper limit normal, *CRS* cytokine release syndrome, *ICANS* immune effector cell-associated neurotoxicity syndrome, *CTCAE* common terminology criteria for adverse events, *n.a.* not applicable, *n.d.* no data

With regard to comorbidities, a higher number of conditions were documented in the CAR T-cell group (Table 2). Cardiovascular diseases were the most prevalent in both groups, followed by metabolic and pulmonary diseases.

**Table 2:** Comorbidities

Organ system	Specific diagnoses	CAR T, *n* (%)	HD-ASCT, *n* (%)
Cardiovascular diseases	Arterial hypertension	11 (73.3)	6 (46.2)
	Atrial fibrillation	4 (26.7)	3 (23.1)
	Heart failure	-	1 (7.7)
	Coronary heart disease	1 (6.7)	1 (7.7)
Metabolic disorders	Thyroid disorders	4 (26.7)	4 (30.8)
	Obesity	2 (13.3)	1 (7.7)
	Type II diabetes mellitus	1 (6.7)	1 (7.7)
Respiratory disease	Thrombosis/pulmonary embolism	2 (13.3)	3 (23.1)
	COPD	2 (13.3)	1 (7.7)
Renal insufficiency	-	1 (6.7)	1 (7.7)
Neurological-psychiatric disorders	Polyneuropathy	2 (13.3)	-
	Stroke	-	1 (7.7)
	Epilepsy	-	1 (7.7)
	Early brain damage	-	1 (7.7)
	Depression	-	1 (7.7)
Others	Rheumatic diseases	2 (13.3)	-
	Post-liver transplant	1 (6.7)	-

Abbreviations: *COPD* chronic obstructive pulmonary disease

### Treatment related toxicities

Following CAR T-cell therapy CRS occurred in 93% of patients (all grade 1–3), and ICANS occurred in 20% (all grade 1, except one grade 3) (Table 1). 60% of patients (*n* = 9) required tocilizumab due to CRS. Infectious complications during initial hospitalization were significantly more frequent in HD-ASCT patients (92% vs. 20% in CAR T, *p* < 0.001). HD-ASCT recipients developed acute grade ≥ 3 toxicities like mucositis (50%), diarrhea (29%), and skin toxicity (14%), none of which were observed in CAR T-cell patients. Median initial hospital stay was shorter for CAR T-cell with 22 days (IQR 8.5) and 29 days for HD-ASCT recipients (IQR 10).

Hematological toxicity (Table 3) was observed in all HD-ASCT patients and in 10 patients (67%) in the CAR T-cell group. All HD-ASCT patients developed severe neutropenia and thrombocytopenia. 92% (*n* = 12) of HD-ASCT patients had severe anemia. Long-term hematotoxicity following CAR T-cell therapy was detected in 8 patients (53%) with prolonged neutropenia, six patients (40%) with severe and prolonged thrombocytopenia, and seven patients (47%) with severe and prolonged anemia. The median time to recovery of all three hematopoietic cell lineages for both cohorts are shown in Table 3 and were each significantly shorter in favor of the HD-ASCT group.

**Table 3:** Therapy-associated hematotoxicity

	CAR T	HD-ASCT	*p*-value
Severe neutropenia^a^, n (%)	10 (67)	13 (100)	n.a.
Prolonged neutropenia^b^, n (%)	8 (53)	-	n.a.
Time to neutrophil recovery (days), median (IQR)	62 (14.75)	9 (4)	< 0.001
Severe thrombocytopenia^c^, n (%)	7 (47) Prolonged^d^: 6 (40)	13 (100)	n.a.
Time to platelet recovery (days), median (IQR)	64 (18.5)	12 (9)	0.005
Severe anemia^e^, n (%)	8 (53) Prolonged^f^: 7 (47)	12 (92)	n.a.
Time to hemoglobin recovery (days), median (IQR)	46 (19.5)	14 (11.5)	0.002

Abbreviations:^a^ANC < 500/μl; ^b^ANC < 1000/μl and/or G-CSF dependent ≥ 21 days after CAR T-cell infusion and continuing ≥ 21 days; ^c^platelet counts < 20/nL and/or requiring transfusions; ^d^platelet counts < 20/nL and/or requiring transfusions measured ≥ 21 days after CAR T-cell infusion and continuing for ≥ 21 days; ^e^hemoglobin < 8 g/dL mmol/l and/or requiring transfusions; ^f^hemoglobin < 8 g/dL and/or requiring transfusions measured ≥ 21 days after CAR T-cell infusion and continuing for ≥ 21 days

### QoL assessments

#### EQ-5D-5 L

**Fig. 1:**
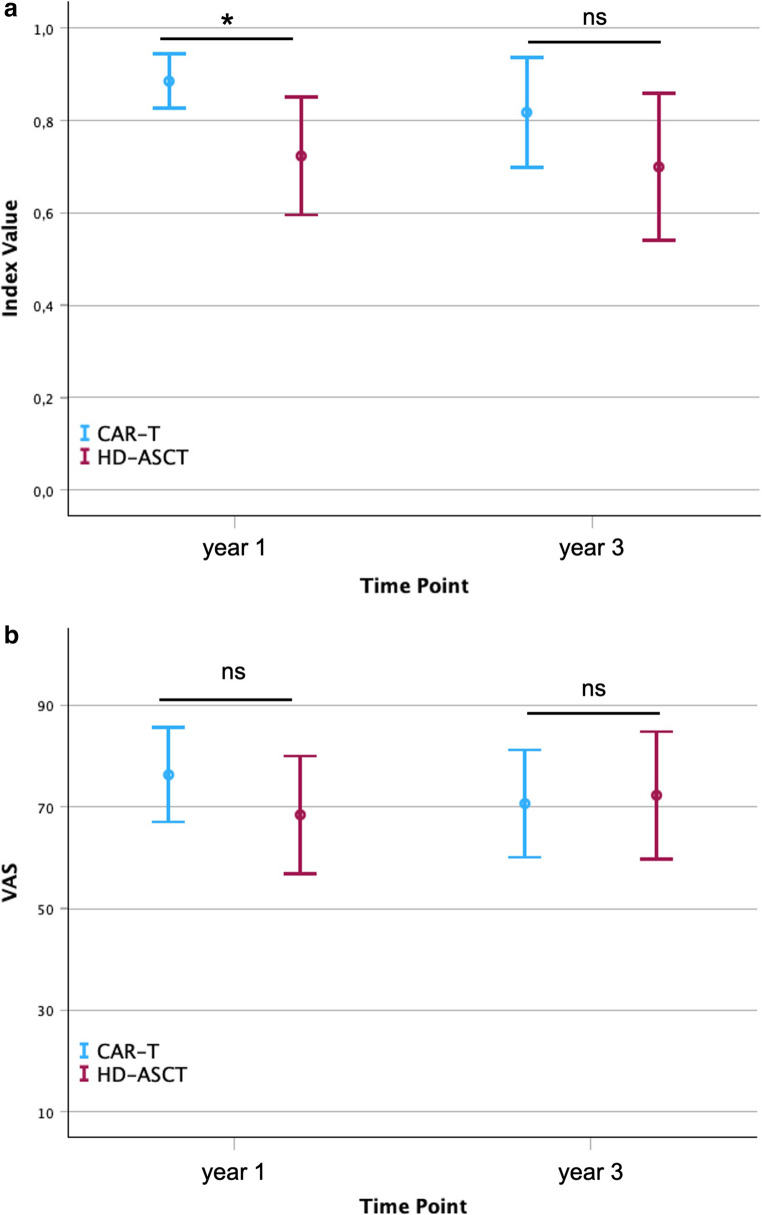
EQ-5D-5 L Index Value and EQ-VAS EQ-5D-5 L Index Value (**A**) and EQ-VAS (**B**) are illustrated for year one and year three *p = 0.015; *ns* not significant (*p* = 0.203)

The frequencies and proportions reported by dimension and level are shown in Table 4, Table 5, and supplemental Tables 1A-1D. The levels “slight problems”, “moderate problems”, “severe problems”, and “extreme problems” are hereafter summarized as “any problems” and contrasted with the level “no problems”. One year following cellular therapy, the majority of patients in the CAR T-cell group reported “no problems” in the categories Mobility (66.7%), Self-Care (73.3%), and Anxiety (66.7%). For Usual Activities and Pain, equal proportions of CAR T-cell patients reported “no problems” (46.7% each) and “any problems” (53.3% each), respectively. In contrast, patients in the HD-ASCT group reported “any problems” in four out of five dimensions, particularly in Usual Activities (92.3%), Anxiety (92.3%), and Pain (84.6%). One year after treatment the mean index value for the CAR T-cell group was 0.89 (± 0.11) vs. 0.72 ± 0.21 for the HD-ASCT group (p = 0.015) (Fig. 1A). Regarding the distribution of responses between the two groups after one year, a significant difference was observed for the dimensions Usual Activities (p = 0.018) and Anxiety (p = 0.01). Three years after therapy, no change in the distribution of responses was observed in the CAR T-cell group for Self-Care and Anxiety (Table 5). However, in the dimensions Mobility (53.3%), Usual Activities (60%), and Pain (73.3%), a higher proportion of CAR T-cell patients now reported “any problems”. Meanwhile, in the HD-ASCT group, a majority of patients continued to report “any problems” in four out of five dimensions. Only in the Self-Care dimension was no change observed from the first time point, and responses remained in favor of level 1 (“no problems”). At year three, no significant difference in the distribution of responses could be demonstrated between the CAR T-cell and HD-ASCT group (Table 5). Similarly, no significant difference in response distributions was observed within either group between the two time points. Three years after cellular therapy, the EQ-5D index value for CAR T-cell and HD-ASCT patients were 0.82 (± 0.22) and 0.70 (± 0.26), respectively (Fig. 1A). This trend still favored CAR T but was no longer statistically significant (p = 0.203).

**Table 4:** EQ-5D-5 L distribution of responses at year 1

Dimension	CAR T year 1 *n* (%)	HD-ASCT year 1 *n* (%)	*p*-value
Mobility			
No problems	10 (66.7)	4 (30.8)	0.141
Slight problems	1 (6.7)	4 (30.8)	
Moderate problems	3 (20.0)	2 (15.4)	
Severe problems	1 (6.7)	3 (23.1)	
Extreme problems	0 (0.0)	0 (0.0)	
Self-Care			
No problems	11 (73.3)	8 (61.5)	0.260
Slight problems	4 (26.7)	2 (15.4)	
Moderate problems	0 (0.0)	2 (15.4)	
Severe problems	0 (0.0)	0 (0.0)	
Extreme problems	0 (0.0)	1 (7.7)	
Usual Activities			
No problems	7 (46.7)	1 (7.7)	0.018
Slight problems	2 (13.3)	8 (61.5)	
Moderate problems	6 (40.0)	3 (23.1)	
Severe problems	0 (0.0)	0 (0.0)	
Extreme problems	0 (0.0)	1 (7.7)	
Pain/Discomfort			
No problems	7 (46.7)	2 (15.4)	0.127
Slight problems	4 (26.7)	3 (23.1)	
Moderate problems	4 (26.7)	8 (61.5)	
Severe problems	0 (0.0)	0 (0.0)	
Extreme problems	0 (0.0)	0 (0.0)	
Anxiety/Depression			
No problems	10 (66.7)	1 (7.7)	0.010
Slight problems	4 (26.7)	6 (46.2)	
Moderate problems	1 (6.7)	5 (38.5)	
Severe problems	0 (0.0)	1 (7.7)	
Extreme problems	0 (0.0)	0 (0.0)

**Table 5:** EQ-5D-5 L distribution of responses at year 3

Dimension	CAR T year 3 *n* (%)	HD-ASCT year 3 *n* (%)	*p*- value
Mobility			
No problems	7 (46.7)	6 (46.2)	0.526
Slight problems	5 (33.3)	2 (15.4)	
Moderate problems	2 (13.3)	2 (15.4)	
Severe problems	1 (6.7)	3 (23.1)	
Extreme problems	0 (0.0)	0 (0.0)	
Self-Care			
No problems	10 (66.7)	6 (46.2)	0.601
Slight problems	2 (13.3)	2 (15.4)	
Moderate problems	2 (13.3)	2 (15.4)	
Severe problems	1 (6.7)	3 (23.1)	
Extreme problems	0 (0.0)	0 (0.0)	
Usual Activities			
No problems	6 (40.0)	3 (23.1)	0.703
Slight problems	5 (33.3)	4 (30.8)	
Moderate problems	2 (13.3)	3 (23.1)	
Severe problems	2 (13.3)	2 (15.4)	
Extreme problems	0 (0.0)	1 (7.7)	
Pain/Discomfort			
No problems	4 (26.7)	4 (30.8)	0.949
Slight problems	5 (33.3)	3 (23.1)	
Moderate problems	5 (33.3)	5 (38.5)	
Severe problems	1 (6.7)	1 (7.7)	
Extreme problems	0 (0.0)	0 (0.0)	
Anxiety/Depression			
No problems	9 (60.0)	3 (23.1)	0.117
Slight problems	5 (33.3)	5 (38.5)	
Moderate problems	1 (6.7)	3 (23.1)	
Severe problems	0 (0.0)	2 (15.4)	
Extreme problems	0 (0.0)	0 (0.0)

No significant difference was found for the EQ-VAS score between CAR T-cell and HD-ASCT patients, either at year one (mean 76.33 ± 16.74 vs. 68.46 ± 19.1; *p* = 0.255) or at year three (mean 70.87 ± 19.07 vs. 72.31 ± 20.68; *p* = 0.829) (Fig. 1B).

#### PROMIS-29

**Fig. 2:**
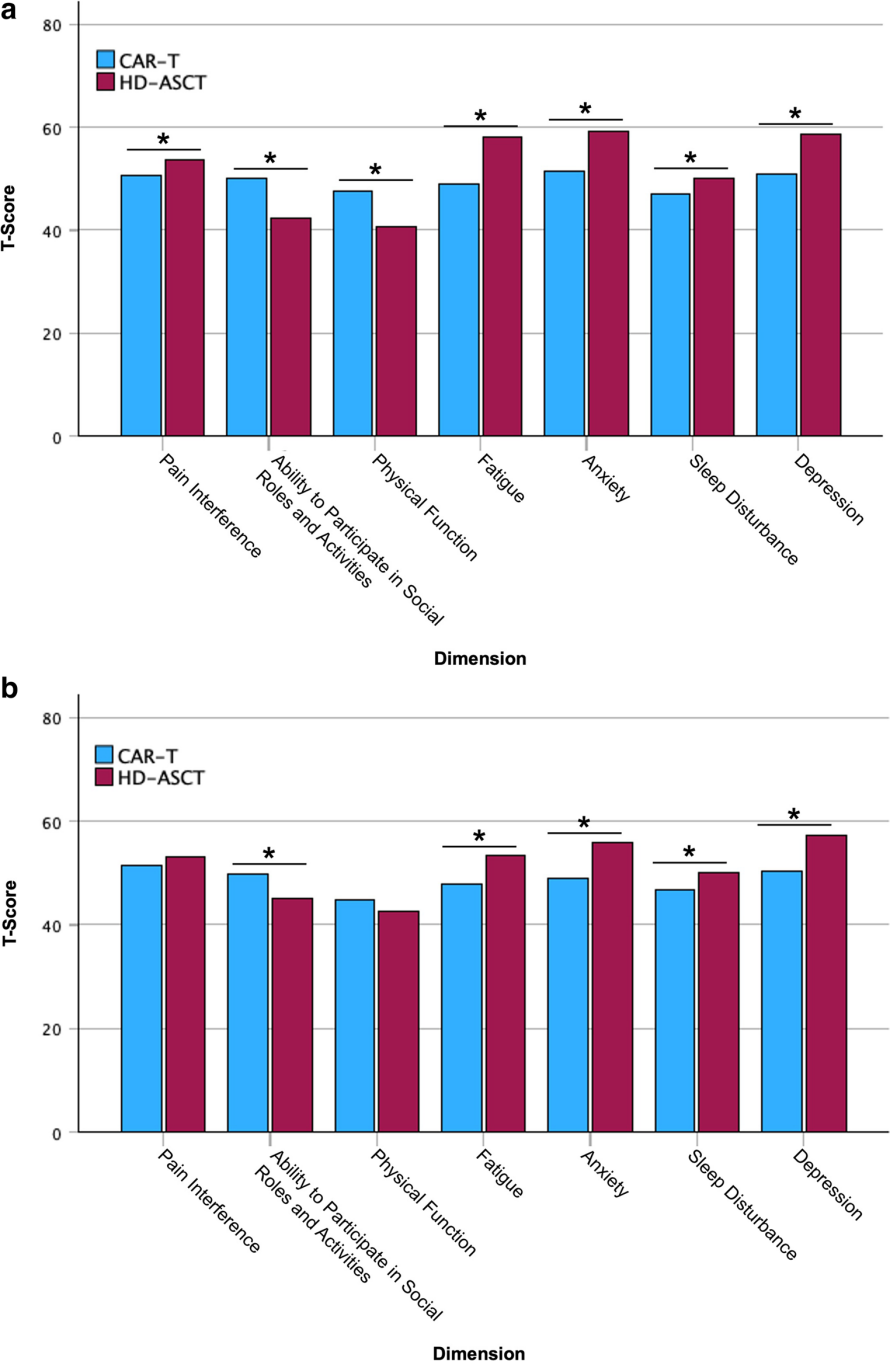
PROMIS-29 T-score for CAR T-cell and HD-ASCT patients are illustrated for year 1 (**A**) and year 3 (**B**). * ≥ 3-point change in T-score

The classification of PROMIS T-scores relative to the general population of the United States is shown in Table 7 for year one and in Table 6 for year three. At year one, the quality of life among CAR T-cell patients was rated as “normal” in all seven domains, whereas HD-ASCT patients reported mild impairment in the domains Social Roles and Activities, Physical Function, Fatigue, Anxiety, and Depression (Table 6). A difference considered clinically significant was defined as a 3-point change in T-score. Based on this criterion, a significant difference between CAR T-cell and HD-ASCT patients was observed in all seven domains at year one, favoring CAR T-cell therapy (Fig. 2A).

**Table 6:** Classification of PROMIS-29 T-scores relative to the general population at year 1

Dimension	CAR T	HD-ASCT
Pain Interference	Normal (50.53)	Normal (53.75)
Ability to Participate in Social Roles and Acivities	Normal (50.09)	Mild (42.4)
Physical Function	Normal (47.44)	Mild (40.54)
Fatigue	Normal (49.09)	Mild (58.01)
Anxiety	Normal (51.54)	Mild (59.22)
Sleep Disturbance	Normal (47.13)	Normal (50.15)
Depression	Normal (50.87)	Mild (58.71)

**Table 7:** Classification of PROMIS-29 T-scores relative to the general population at year 3

Dimension	CAR T	HD-ASCT
Pain Interference	Normal (51.35)	Normal (53.19)
Ability to Participate in Social Roles and Acivities	Normal (49.67)	Normal (45.09)
Physical Function	Mild (44.83)	Mild (42.58)
Fatigue	Normal (47.78)	Normal (53.29)
Anxiety	Normal (48.86)	Mild (55.9)
Sleep Disturbance	Normal (46.79)	Normal (50.12)
Depression	Normal (50.25)	Mild (57.35)

At year three, a decline from “normal” to “mild” was noted in the Physical Function domain among CAR T-cell patients, with a difference of 2.61 points that did not reach statistical significance. In all other six domains, no significant changes in assessment were observed in this group. In the HD-ASCT group, improvements from “mild” to “normal” were observed at year three compared to year one in the domains Social Roles and Activities and Fatigue. Furthermore, within the HD-ASCT group, significant improvements (T-score change > 3) were observed in the domains Fatigue and Anxiety between year one and year three (Table 7). When comparing CAR T-cell and HD-ASCT patients at year three, significant differences in favor of CAR T-cell therapy persisted in the domains Social Roles and Activities, Fatigue, Anxiety, Sleep Disturbance, and Depression. In the domains Pain Interference and Physical Function, results were comparable between the groups (Fig. 2B).

Additionally, pain intensity was assessed using the NRS scale within the PROMIS-29.

No significant differences were observed between CAR T-cell and HD-ASCT patients at either year one (mean 1.87 ± 2.03 vs. 2.62 ± 2.53; *p* = 0.393) or year three (mean 1.93 ± 2.34 vs. 2.46 ± 2.37; *p* = 0.559). Similarly, within the CAR T-cell group, no significant difference was found between year one and year three (mean 1.87 ± 2.03 vs. 1.93 ± 2.34; *p* = 0.872). Likewise, there was no significant difference within the HD-ASCT group between the two time points (mean 2.62 ± 2.53 vs. 2.46 ± 2.37; *p* = 0.760).

## Discussion

Therapeutic decisions are no longer made solely based on response and survival duration, but increasingly also take into account patients’ QoL [22]. In the present study, which was primarily designed to provide descriptive insights into HRQoL after cellular therapy, it has been shown that for rr LBCL patients in sustained remission, CAR T-cell therapy was associated with superior HRQoL compared to HD-ASCT at one year after treatment, and in the long-term follow-up. These results, derived from a homogeneous patient cohort with uniform treatment protocols, add to the growing evidence favoring CAR T from a patient-centered outcomes perspective [10, 14]. A key strength of this analysis is the long observation period with a median of 36.8 months. Very few studies have observation periods of ≥ 24 months, as the number of patients remaining in long-term remission declines over time due to relapse or death, resulting in limited data for extended follow-up periods [9]. In our analysis, it should be acknowledged that baseline differences existed between the CAR T-cell and HD-ASCT groups. CAR T-cell patients were significantly older and more heavily pretreated with significantly higher number of prior therapies (median prior lines of therapy: 4, IQR 1 vs. 2, IQR 0; *p* < 0.001). Moreover, nearly half of the CAR T-cell patients had previously undergone HD-ASCT, reflecting a more intensive pretreatment. Additionally, CAR T-cell patients had a worse median ECOG performance status at the time of cellular therapy (1, IQR 1 vs. 0, IQR 1), which can be partially attributed to a more intensive disease history, and had a higher number of comorbidities. Yet, despite representing a higher-risk group, CAR T-cell recipients had better HRQoL outcomes than HD-ASCT patients. The overall lower toxicity profile of CAR T compared to HD-ASCT enables older and less fit patients to be treated with manageable side effects [23, 24].

Recovery of HRQoL after CAR T-cell therapy seem to proceed faster than after HD-ASCT. Although CAR T-cell therapy is associated with a nadir in quality of life during the first 4 weeks due to side effects such as CRS and ICANS, which vary in frequency depending on several factors (e.g. disease status at CAR T-cell infusion, inflammatory state, CAR T-cell product), the patients already return to baseline QoL levels after 4 weeks. After 6 months, patients report even better QoL than before therapy. In contrast, patients treated with HD-ASCT experience a nadir between day 30 and 100 and return to baseline QoL only after 3–6 months. In the long-term follow-up, a stable but not superior HRQoL is observed in the HD-ASCT group [14].

In the direct comparison between CAR T-cell therapy and HD-ASCT, we observed a significantly better HRQoL in favor of CAR T-cell therapy, both in the EQ-5D-5 L and PROMIS-29 questionnaires. At year one after cellular therapy, this was reflected by a significantly higher index value in the EQ-5D-5 L and a significantly different distribution of responses in the domains Usual Activities and Anxiety, where CAR T-cell patients more frequently reported “no problems”. Accordingly, the mean value for the EQ-VAS in the CAR T-cell group was higher at year one, but the difference was not statistically significant.

In the PROMIS-29, all seven domains assessed in the CAR T-cell group were comparable to the general population and differed significantly from those of the HD-ASCT group (defined as a 3-point variation in T-score). These results underline the better tolerability and lower toxicity profile of CAR T-cell therapy despite CRS and ICANS within the first year after therapy. The lower toxicity profile is also reflected in a shorter hospital stay of CAR T-cell patients compared to HD-ASCT patients.

Severe chemotherapy-associated side effects such as mucositis, diarrhea, or skin toxicity following HD-ASCT did not occur in the CAR T-cell group, as the preceding lymphodepleting chemotherapy is less intensive and therefore better tolerated. Nevertheless, severe hematotoxicity was observed after both therapies, affecting all HD-ASCT patients and 67% of CAR T-cell patients across all three cell lines. However, significantly fewer CAR T-cell patients experienced severe infectious complications during hospitalization compared to HD-ASCT patients. Notably, hematological reconstitution after CAR T-cell therapy took significantly longer for all three cell lines, which can be attributed to immunological effects of CAR T-cells on hematopoietic stem cells in the bone marrow [25], inflammation-mediated toxicity [26], and the higher number of prior therapies, especially previous HD-ASCT [27]. Despite prolonged hematotoxicity after CAR T-cell therapy, this did not appear to substantially affect HRQoL one year after therapy, as all three cell lines - despite single cases of very prolonged neutropenia - recovered within a median of 60 days.

At year three following cellular therapy, the EQ-5D-5 L results converged between the two patient groups, and differences were no longer significant. In the PROMIS-29, however, the results for CAR T-cell patients remained stable with the longer follow-up in all domains compared to year one, while HD-ASCT patients reported clinically relevant improvements in fatigue and anxiety. Nevertheless, a significant superior HRQoL remained in five of the seven domains in favor of CAR T-cell therapy.

Regarding pain, which was additionally assessed using the NRS scale within the PROMIS-29, only very low pain levels were reported, and there were no significant differences between the groups or the two time points. The consistently low pain levels without relevant dynamics might be explained by the persistent long-term remission and the absence of disease-related pain.

In summary, our data suggest that patients with rr LBCL who receive CAR T-cell therapy experience faster functional, physical, and psychological recovery compared to those treated with HD-ASCT, despite being older and having undergone more intensive pretreatment. CAR T-cell therapy is associated with fewer or even absent severe concomitant symptoms such as infections, pain, mucositis, or diarrhea. The CAR T-specific side effects CRS and ICANS impair patients’ HRQoL particularly in the first 2–4 weeks after therapy [14], but do not appear to have a more severe long-term impact than the side effects of HD-ASCT. The lower rate of complications and symptoms also seems to allow shorter hospitalization and a faster return to home life. Our data further suggest that a lower burden of physical symptoms after CAR T-cell therapy subsequently leads to less stress, anxiety, and depression, thereby possibly accelerating psychological recovery. However, late toxicities such as hematologic and solid secondary malignancies are a known risk of CAR T-cell therapy and monitoring for late complications is essential to improve the outcomes and HRQoL [28]. Given the limited sample of 28 patients in total and the observational design, the generalizability of our findings may be restricted. This study highlights the clinical relevance of PROs in rr LBCL, particularly in longitudinal surveys. However, larger prospective studies are warranted to assess the long-term effects of these therapies on HRQoL as the integration of patient-centered metrics into therapeutic decision-making and long-term care planning is becoming increasingly important.

## Supplementary Information

Below is the link to the electronic supplementary material.

Supplementary Material 1 (PDF 43.3 KB) 

## Data Availability

No datasets were generated or analysed during the current study.
