# Supplementary material for: Tisagenlecleucel yields superior patient-reported health-related quality of life compared to autologous stem cell transplantation in patients with relapsed/refractory large B-cell lymphomas
Source: Ann Hematol. 2026 Feb 5;105(3):88. doi: 10.1007/s00277-026-06840-5 (PMC12872639; doi:10.1007/s00277-026-06840-5)
Supplement: Supplementary file 1 — Supplementary Material 1 (PDF 43.3 KB) [file 277_2026_6840_MOESM1_ESM.pdf]

Supplemental Table 1A EQ-5D-5L frequencies and proportions for CAR T-cell group at year 1

CAR T<br>Time Point 1	Mobility,<br>n (%)	Self-Care,<br>n (%)	Usual Activities,<br>n (%)	Pain / Discomfort,<br>n (%)	Anxiety / Depression,<br>n (%)
**Level 1<br>(no problems)**	10 (66.7)	11 (73.3)	7 (46.7)	7 (46.7)	10 (66.7)
**Level 2-5<br>(any problems)**	5 (33.4)	4 (26.7)	8 (53.3)	8 (53.3)	5 (33.4)
**TOTAL**	15 (100)	15 (100)	15 (100)	15 (100)	15 (100)

Supplemental Table 1B EQ-5D-5L frequencies and proportions for CAR T-cell group at year 3

CAR T<br>Time Point 2	Mobility,<br>n (%)	Self-Care,<br>n (%)	Usual Activities,<br>n (%)	Pain / Discomfort,<br>n (%)	Anxiety / Depression,<br>n (%)
**Level 1<br>(no problems)**	7 (46.7)	10 (66.7)	6 (40.0)	4 (26.7)	9 (60.0)
**Level 2-5<br>(any problems)**	8 (53.3)	5 (33.4)	9 (60.0)	11 (73.3)	6 (40.0)
**TOTAL**	15 (100)	15 (100)	15 (100)	15 (100)	15 (100)

Supplemental Table 1C EQ-5D-5L frequencies and proportions for HD-ASCT group at year 1

HD-ASCT<br>Time Point 1	Mobility,<br>n (%)	Self-Care,<br>n (%)	Usual Activities,<br>n (%)	Pain / Discomfort,<br>n (%)	Anxiety / Depression,<br>n (%)
**Level 1<br>(no problems)**	4 (30.8)	8 (61.5)	1 (7.7)	2 (15.4)	1 (7.7)
**Level 2-5<br>(any problems)**	9 (69.2)	5 (38.5)	12 (92.3)	11 (84.6)	12 (92.3)
**TOTAL**	13 (100)	13 (100)	13 (100)	13 (100)	13 (100)

Supplemental Table 1D EQ-5D-5L frequencies and proportions for HD-ASCT group at year 3

HD-ASCT Time Point 2	Mobility, n (%)	Self-Care, n (%)	Usual Activities, n (%)	Pain / Discomfort, n (%)	Anxiety / Depression, n (%)
Level 1 (no problems)	6 (46.2)	8 (61.5)	3 (23.1)	4 (30.8)	3 (23.1)
Level 2-5 (any problems)	7 (53.8)	5 (38.5)	10 (76.9)	9 (69.2)	10 (76.9)
TOTAL	13 (100)	13 (100)	13 (100)	13 (100)	13 (100)
